# Interactive simulator for e-Learning environments: a teaching software for health care professionals

**DOI:** 10.1186/1475-925X-13-172

**Published:** 2014-12-18

**Authors:** Claudio De Lazzari, Igino Genuini, Domenico M Pisanelli, Alessandra D’Ambrosi, Francesco Fedele

**Affiliations:** CNR, Institute of Clinical Physiology, UOS of Rome, Via S.M. della Battaglia, 44, 00185 Rome, Italy; National Institute for Cardiovascular Research, Bologna, Italy; Department of Cardiovascular, Respiratory, Nephrological, Anesthesiological and Geriatric Sciences, “Sapienza” University of Rome, Rome, Italy; CNR, Institute of Cognitive Sciences and Technology (ISTC), Rome, Italy

**Keywords:** e-Learning, Numerical model, Cardiovascular system, Thoracic artificial lung assistance, Haemodynamic, Ontologies

## Abstract

There is an established tradition of cardiovascular simulation tools, but the application of this kind of technology in the e-Learning arena is a novel approach. This paper presents an e-Learning environment aimed at teaching the interaction of cardiovascular and lung systems to health-care professionals. Heart-lung interaction must be analyzed while assisting patients with severe respiratory problems or with heart failure in intensive care unit. Such patients can be assisted by mechanical ventilatory assistance or by thoracic artificial lung.

“*In silico*” cardiovascular simulator was experimented during a training course given to graduate students of the School of Specialization in Cardiology at ‘Sapienza’ University in Rome.

The training course employed CARDIOSIM^©^: a numerical simulator of the cardiovascular system. Such simulator is able to reproduce pathophysiological conditions of patients affected by cardiovascular and/or lung disease. In order to study the interactions among the cardiovascular system, the natural lung and the thoracic artificial lung (TAL), the numerical model of this device has been implemented. After having reproduced a patient’s pathological condition, TAL model was applied in parallel and hybrid model during the training course.

Results obtained during the training course show that TAL parallel assistance reduces right ventricular end systolic (diastolic) volume, but increases left ventricular end systolic (diastolic) volume. The percentage changes induced by hybrid TAL assistance on haemodynamic variables are lower than those produced by parallel assistance. Only in the case of the mean pulmonary arterial pressure, there is a percentage reduction which, in case of hybrid assistance, is greater (about 40%) than in case of parallel assistance (20-30%).

At the end of the course, a short questionnaire was submitted to students in order to assess the quality of the course. The feedback obtained was positive, showing good results with respect to the degree of students’ learning and the ease of use of the software simulator.

## Background

The e-Learning industry is developing at a fast pace. In the medical context, in particular, it is going to offer “face to face” classes, like those proposed in university rooms. Unfortunately tutors mainly employ static contents (e.g. PowerPoint or Word files or Virtual Learning Environments (VLEs) or websites). Such contents require no real interaction by the users [1]. Simulators can increase the degree of interaction in health care education, for example by realizing a “virtual patient”, implemented by means of interactive computer simulations [1–3]. In this way the learner will take profit of a various range of learning activities. Other notable experiences include for instance Collaborative Tutoring Systems to determine clinical-reasoning skills of students [4].

In this paper we present our experience of an e-Learning course realized using the numerical simulator of the cardiovascular system CARDIOSIM^©^ [5]. This interactive software is able to reproduce physiological and pathophysiological conditions of patients affected by cardiovascular and/or lung disease [6, 7]. In the simulator, several different ventricular pumps and devices was implemented in order to assist one or both ventricles in parallel or in series [8–11]. With respect to previous CARDIOSIM^©^ version, thoracic artificial lung (TAL) assistance simulation was implemented too. This new version is also able to reproduce the cardiopulmonary pathological conditions of a patient and to study the interaction among the cardiovascular system, the natural lung and the TAL.

During a training course held for graduate students of the School of Specialization in Cardiology at the Department of Cardiovascular, Respiratory, Nephrological, Anesthesiological and Geriatric Sciences (hereinafter CRNAGS Department), “Sapienza” University of Rome, heart-lung interaction was studied when thoracic artificial lung assistance was applied.

Heart-lung interaction must be analyzed when patients with heart failure or with severe respiratory problems are assisted in Intensive Care Cardiac Unit. Some pulmonary diseases can be the result of haemodynamic pathology, most notably an increase in pulmonary vascular resistance. In these cases the right ventricle (RV) is overloaded and cardiac output is reduced to problematic levels. In many pulmonary diseases, both gas exchange and haemodynamic disturbances are present to some degree. Therefore an accurate analysis of the heart-lung interaction must be done in those patients with severe respiratory problems or with heart failure [12].

Chronic respiratory failure or severe pulmonary infections can be treated using thoracic artificial lung that do not use a pump [13] but rather can be attached to the natural lung (NL) in series, in parallel or in hybrid mode [14].

During the course the choice of patients used for reproducing pathological conditions has been done using on a set of pathological clinical patients. In these pathological conditions (the baseline conditions for students who start the interaction with the simulation tool), it is possible to observe the following effects: an increase of right ventricular end systolic volume, an increase of right ventricular end diastolic volume and an important increase of pulmonary arterial pressure. Starting from baseline conditions, students have been trained to manage the virtual patient’s pathology by acting in different ways. At first, students applied parallel TAL assistance and set pulmonary arterial peripheral resistance to high value in order to induce a pathological overload in the right ventricle. In a second moment, the same simulations were performed setting pulmonary arterial peripheral resistance to physiological value in order to simulate the effects induced by drugs that permit to download the work of the right ventricle. Finally students reproduced the same simulations using the hybrid TAL assistance. Results obtained by student’s simulations showed that parallel TAL assistance:

- increases both left ventricular and end systolic and end diastolic volume;
- reduces right ventricular and end systolic and end diastolic volume;
- reduces mean pulmonary arterial pressure.

All students’ simulations have been performed in an interactive way. By interacting with the tool, students were able to put into practice concepts learned during their previous studies. Sometime they made mistakes that have been highlighted by the simulator. By simulating an inappropriate drug administration, students have been able to verify how the conditions of the virtual patient worsened.

During our experience, it has been proved that cardiovascular interactive software simulator can be a useful tool for studying and training cardiologists, anaesthesiologists, nurses and medical graduate and undergraduate students in the management of mechanical circulatory and ventilatory devices [15–17].

## Materials and methods

The software simulator developed by the Institute of Clinical Physiology of the Italian National Research Council is able to reproduce pathophysiological conditions of patients affected by cardiovascular and/or lung diseases. The simulator implements many different cardiovascular pumps in order to assist one or both ventricles in parallel or in series way [6, 8–10]. The blood pump implemented can reproduce pulsatile or continuous flow.

The lung assistance is realized by a new software module implementing a thoracic artificial lung. TAL can be connected to the NL in parallel, in series and in hybrid mode [14, 18, 19]. CARDIOSIM^©^ has a graphical and a numerical interface which shows the instantaneous waveforms during the cardiac cycle and the mean values of pressures, volumes and flow.

The training course, held for graduate students, consisted of different exercises in which the pathophysiological conditions of patients hospitalized in CRNAGS Department were reproduced using the interactive software simulator.

**Figure 1:**
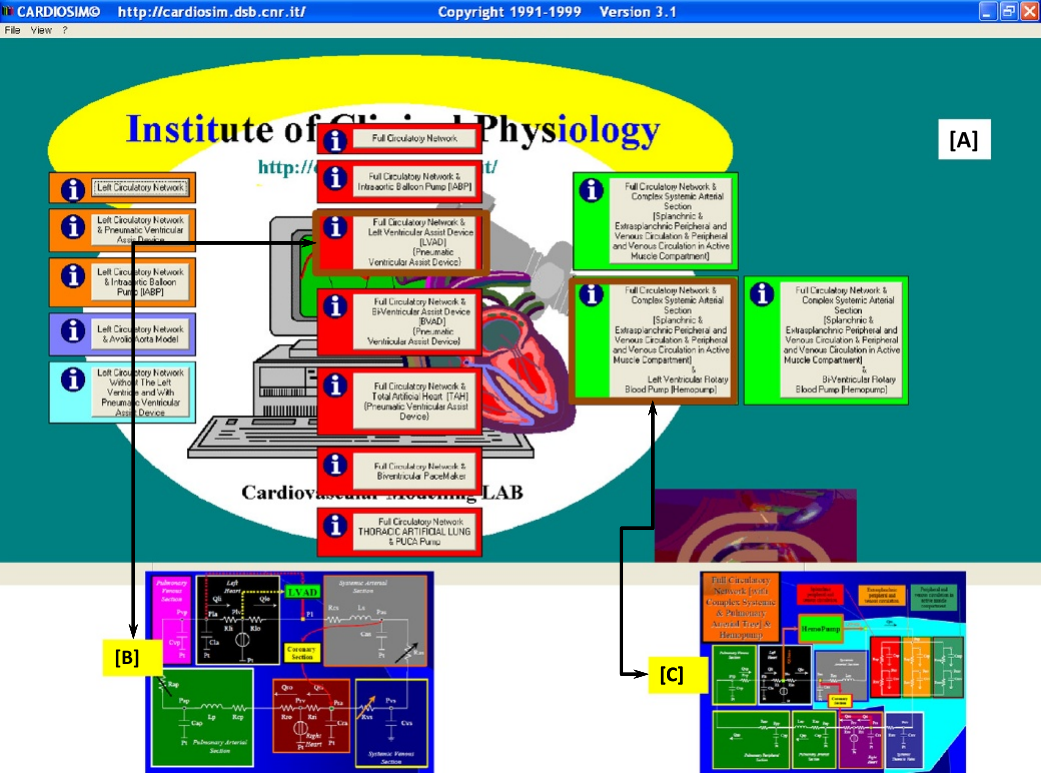
**Software interface used during the e**-**Learning training course.** Window **[A]** represents a first level of choice that permits to select among different districts of cardiocirculatory network, different mechanical assist devices and different mechanical ventilatory assistance. Panels **[B]** and **[C]** show two different possible networks choose. In panel **[B]** is represented a network with a simple implementation of systemic and pulmonary arterial sections and with a parallel pneumatic left ventricular assist device (LVAD). In panel **[C]** is represented a network with a complex implementation of systemic and pulmonary arterial tree and with “in series” rotary left ventricular assist device (Hemopump).

In this paper we describe our experience gained during the simulations cycle held in a classroom with 25 students. Each pair of students was equipped with a workstation on which the CARDIOSIM^©^ software was installed. The exercises were focused on the study of the effects induced on some haemodynamic variables by the heart-lung interaction in presence of TAL assistance. Figure 1 (panel A) shows the software interface used during the e-Learning course relative to a first level of choice that permits to select different districts of cardiocirculatory network, different mechanical assist devices and different mechanical ventilatory assistance. Panels B and C show two different possible networks choose. Starting from baseline conditions, reproducing the pathological conditions of hospitalized patient, the effects of different TAL assistances (in parallel and hybrid mode) were reproduced by changing TAL compliances and resistances [13, 14], in presence of different pulmonary vascular resistance values. The selection of patients undergoing this type of therapy had been previously carried out by the students on the basis of an ontology based on the evaluation of measured haemodynamic parameter values.

### The cardiovascular model

**Figure 2:**
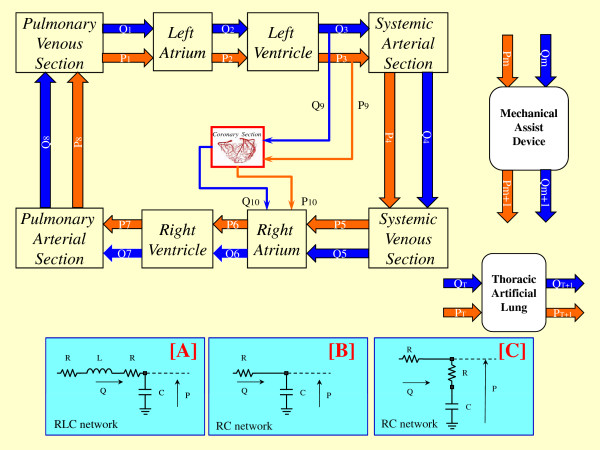
**Overall modular configuration of the software simulator.** Each block of the circulatory network, enclose “Mechanical Assist Device” and “Thoracic Artificial Lung” blocks, can be implemented as a combinations of “*n*” different sub-blocks. The generic block (or sub-block) can be implemented using different combinations of the three electrical networks **A**, **B**, or **C** realised by resistance (R), inductance (L) and compliance (C). Each block (or sub-block) has two inputs and two outputs representing flow (Q) and pressure (P).

The overall modular configuration of the software simulator is reported in Figure 2. Each block of the circulatory network can be implemented as combinations of “*n*” different sub-blocks. The generic block (or sub-block) can be implemented using different combinations of the three electrical networks reported in figure. Each block (or sub-block) has two inputs and two outputs representing flow (Q) and pressure (P). Panels B, C of Figure 1 are two possible circulatory network representations obtained starting by the configuration presented in Figure 2. In this figure also mechanical assist device (MAD) and thoracic artificial lung are represented as block having two inputs and two outputs. This kind of assistances can be connected to the circulatory network in different way (e.g. MAD can be connected in series or in parallel to both ventricles). All single sub-blocks of the network can be implemented using one of the three electrical representations realised by resistance (R), inductance (L) and compliance (C). Each sub-block can be implemented numerically by first-order partial differential equations. In the software simulator, the entire equation system is solved by Euler’s method.

**Figure 3:**
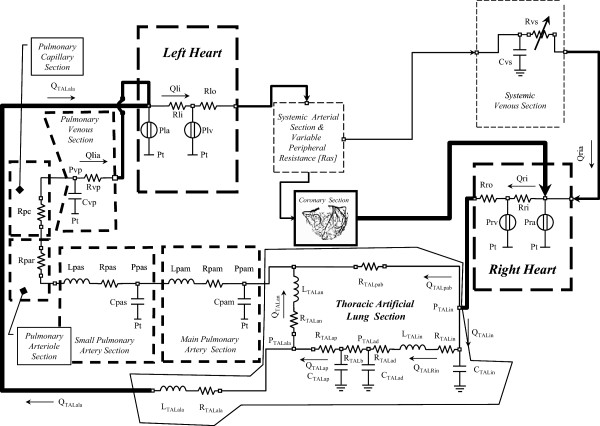
**Electrical analog model of the cardiovascular simulator including the thoracic artificial lung.** The cardiovascular model is divided in the following sections: left (right) heart, systemic arterial section (with variable peripheral resistance), systemic venous section, main (small) pulmonary artery section, pulmonary arteriole (capillary) section, pulmonary venous section and coronary circulation. TAL assistance is connected between the input to the pulmonary artery and the natural lung/left atrial section. Table 1 summarizes pressures, flows and RLC elements.

CARDIOSIM^©^ configuration used during the course held for the graduate students of the CRNAGS Department is reported in Figure 3 (Table 1). The electrical analogue of the cardiovascular system (CVS) shows the left and right ventricles (atrium), coronary section, systemic arterial and venous sections, main and small pulmonary artery sections, pulmonary arteriole and capillary sections, pulmonary venous section and thoracic artificial lung section.

**Table 1:** **Legends of parameters and variables used in CARDIOSIM** ^**©**^ **computer simulator**

Symbols	Variables/parameters	Symbols	Variables/parameters
*Pla* (*Pra*)	Left (Right) atrial pressure	*Rpar*(*Rpc*)	Pulmonary arteriole (capillary) resistance
*Plv* (*Prv*)	Left (Right) ventricular pressure	*Rvp*	Pulmonary venous resistance
*Pvs*	Systemic venous pressure	*Ras*	Systemic peripheral arterial resistance
*Ppam*	Main pulmonary artery pressure	*Cvs*(*Cvp*)	Systemic (Pulmonary) venous compliance
*Ppas*	Small pulmonary artery pressure	*Cpam*(*Cpas*)	Main (Small) pulmonary artery compliance
*Pvp*	Pulmonary venous pressure	*LTALin*, *LTALala*, *LTALan*	The three inductances model the inertia in the grafts. They depend by the blood density, the graft radius and the graft length
*Pt*	Mean intrathoracic pressure	*Lpam*(*Lpas*)	Inertance of main (small) pulmonary artery
*Qlia*(*Qria*)	Left (Right) atrial input flow	*Cvs*(*Cvp*)	Systemic (Pulmonary) venous compliance
*Qli*(*Qri*)	Left (Right)ventricular input flow	*CTALad*(*CTALap*)	Compliance of the TAL inlet (outlet) chamber
*Qlo*(*Qro*)	Left (Right) ventricular output flow	*CTALin*	Inlet graft compliance to the TAL
*Rli*(*Rri*)	Left (Right) input valve resistance	*RTALin*	Inlet graft resistance to the TAL
*Rlo*(*Rro*)	Left (Right) output valve resistance	*RTALpab* (*RTALala*)	Resistance used to divide the flow between pulmonary artery (RTALpab) and left atrium (RTALala)
*Rvs*	Systemic venous resistance	*RTALad*(*RTALap*)	Flow dependent inlet (outlet) resistance
*Rpam*(*Rpas*)	Main (Small) pulmonary artery resistance	*RTALan*	Outlet graft resistance to the pulmonary artery

The behaviour of both ventricles, atrium and septum, were reproduced by means of variable elastance model [20–23]. The atrial septum is assumed to be rigid. This representation allows reproducing the Starling’s law of the heart [24]. The different sections of the circulatory network were implemented using lumped parameter models:

- Systemic arterial section was reproduced using a modified windkessel with a variable peripheral resistance [6, 25].
- Systemic venous section was implemented by compliance and variable resistance [7, 9, 25].
- Main and small pulmonary artery sections were reproduced by RLC model [14, 26, 27].
- Pulmonary arteriole and capillary sections were modelled by a single resistance [14].
- Pulmonary venous section was reproduced by RC element [26, 27].
- The behaviour of the coronary network was implemented by lumped parameter model based on the intramyocardial pump concept [11, 28–30].

### The thoracic artificial lung model

**Figure 4:**
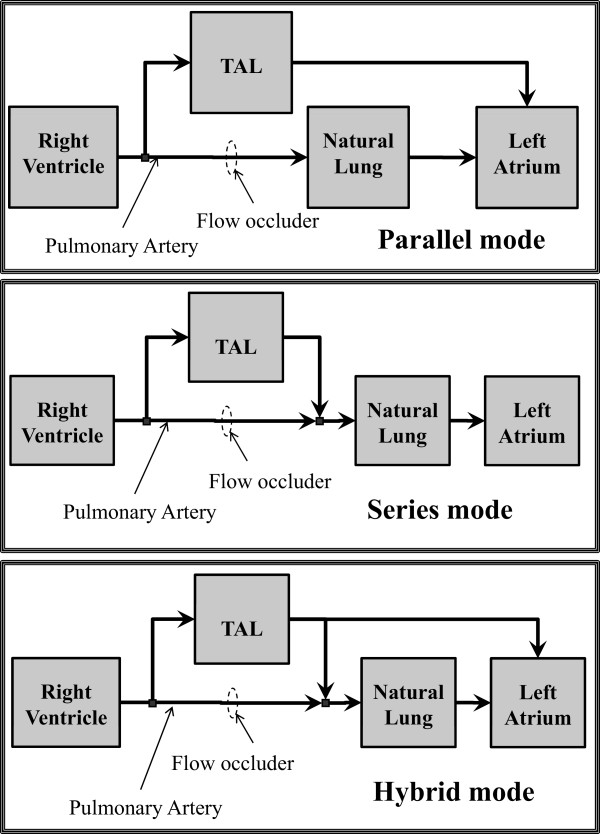
**Shows the possible TAL connections**: **parallel mode** **(upper panel),** **in series mode** **(central panel)** **and hybrid mode** **(lower panel)** **.** In all connections TAL assistance takes blood from pulmonary artery. In parallel (in series) mode TAL ejects blood into left atrium (natural lung). Finally in hybrid connection TAL ejects blood in both natural lung and left atrium. In this last connection the amount of blood ejected into the natural lung depends from the value of resistance R_TALpab_ (Figure 3).

The electrical analogue of the new module implemented into CARDIOSIM^©^ software is shown in Figure 3. TAL assistance can be connected to NL in “series mode”, in “parallel mode” or in “hybrid mode” (Figures 3 and 4) [14, 18, 19]. In “parallel mode” the blood flow is routed from the pulmonary artery through the TAL and then returned to the left atrium (Figure 4). This configuration reduces impedance of the TAL and natural lung system. In “series mode”, the blood flow is routed from the proximal pulmonary artery through the TAL and then back to the distal pulmonary artery, where the blood then flows through the NL and finally back to the left atrium (Figure 4). In “hybrid mode” the blood flow is routed in part to the NL through the TAL and in part to the left atrium through the TAL (Figure 4).

In the numerical simulator, TAL was implemented using a lumped parameter model (Figure 3). Five parameters (R_TALap_, R_TALad_, L_TALap_, L_TALad_ and R_TALb_) were used to model the TAL assistance. Hybrid TAL configuration is realized connecting the pulmonary circulation through the resistance (R_TALpab_) and linking the left atrium through the RL elements (R_TALala_ and L_TALala_). In “series mode” was implemented setting the resistances R_TALpab_ and R_TALala_ to infinity value. Parallel TAL configuration was realised setting the resistance R_TALan_ to infinity value.

### Software interface simulator

**Figure 5:**
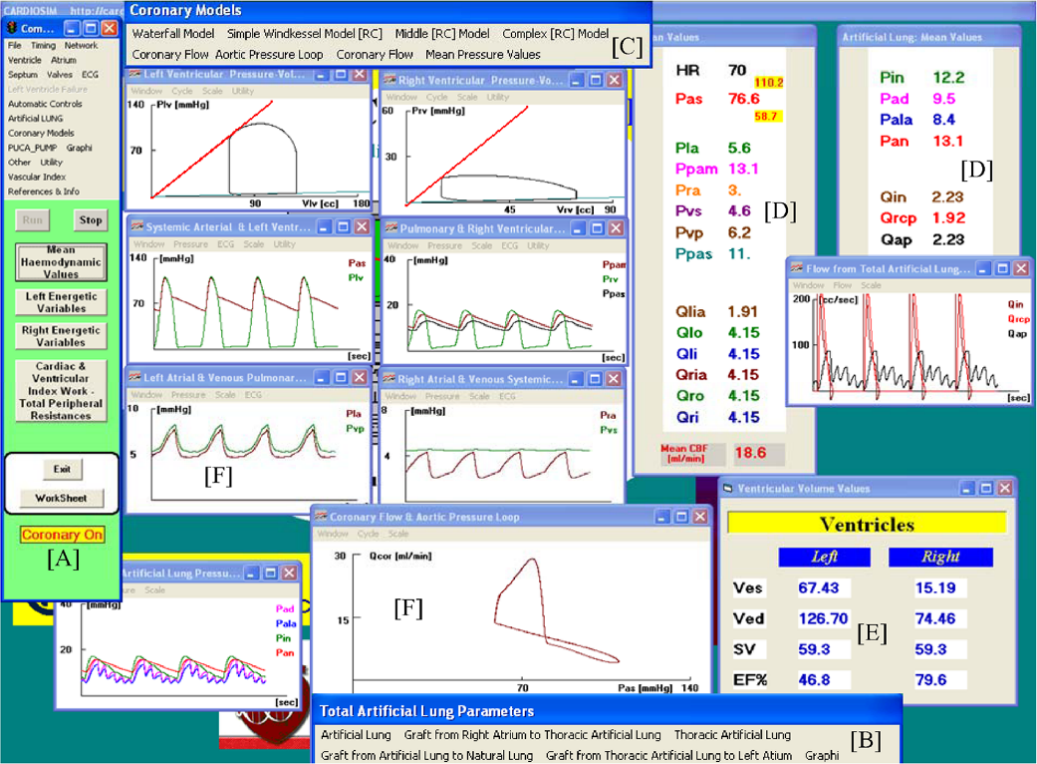
**Interface windows presented to the students during some phases of an exercise which was held during the training course.** Panel **A** represents the “principal” command box, panel **B** the “thoracic artificial lung” command box and panel **C** the “coronary model” command box. Users can perform a second level of choice for the representation of some circulatory districts from panel **A**. In addition using this command box it is possible changes network, ventricular and atrium parameters, it is possible choose different graphical representations and to store parameters and numerical waveforms values. Panel **B** presents commands to set “ON/OFF” TAL assistance, to change TAL configuration and its parameter values and to show the waveforms of pressure and flow of the TAL. Finally panel **C** presents commands to set “ON/OFF” four different representation of coronary circulation. Coronary network is assembled according to the same principle described in Figure 2. Panel **C** permits to change coronary parameter values and to show pressure and flow waveforms in different coronary districts. Panels **D** and **E** show the mean pressure, volume and flow values. In all other windows **(F)** the instantaneous pressure, volume and flow waveforms calculated in the various districts of the network are plotted.

Figure 5 shows the interface windows presented to students during some phases of a tutorial in which a simulated pathological patient was assisted by means of a TAL device. The figure shows some of the possible “command boxes” (e.g. “principal” command box (panel A) in the left side, “thoracic artificial lung” (panel B) and “coronary model” (panel C) command boxes. In “principal” command box (panel A), users can perform a second level of choice for the representation of some circulatory districts. In addition, by using this box it is possible to change network, ventricular and atrium parameters, and also to choose different graphical representations and to store parameters and numerical waveforms values. “Thoracic artificial lung” box (panel B) presents commands to set “ON/OFF” TAL assistance, to change TAL configuration (parallel, in series and hybrid) and its parameter values and to show the waveforms of pressure and flow of the TAL. Finally “coronary model” box (panel C) presents command to set “ON/OFF” four different models of coronary circulation. Coronary network is assembled according to the same principle described in Figure 2. This last command box (panel C) permits to change coronary parameter values and to show pressure and flow waveforms in different coronary districts. Panels D and E (Figure 5) show mean pressure, volume and flow values calculated during the cardiac cycle.

In the different windows (F) the instantaneous pressure, volume and flow waveforms in correspondence of different cardiocirculatory districts are plotted.

## Experimental method

The training course on the interaction of cardiovascular and lung systems was held for 25 graduate students of the School of Specialization in Cardiology and consisted in 8 two-hour sessions. During such sessions, cardiocirculatory pathological conditions of patients have been reproduced. The reproduced conditions were comparable with those measured on the patients analyzed and described in our previous papers [7, 10, 11]. The patients selection were done on the ontological concept basis applied on haemodynamic measured variables. By using the numerical simulator, the students simulated various drug therapies aimed at improving the patients’ cardiocirculatory conditions [7]. The training course took into account also pathological cardiocirculatory conditions which, in order to be solved, need: ventricular assist devices (VADs) [6, 8, 9], intra-aortic balloon pump (IABP) [31], biventricular pacemakers [10], mechanical ventilatory assistance [8, 9] and so on. In these kinds of exercises students use ontology in order to choose the mechanical circulatory support systems which best suits to solve the simulated patient’s pathology [32].

In the following we describe a case study carried out during the seminars. Using data measured on patients (admitted to the CRNAGS Department), students reproduced a pathological condition that induced the following effects on the haemodynamic variables: an increase of right ventricular end systolic volume, an increase of right ventricular end diastolic volume and an important increase of pulmonary arterial pressure.

Starting by these circulatory conditions (baseline conditions), the experiments were divided in different steps. In the first step parallel TAL assistance was applied, and the pulmonary arterial peripheral resistance was set to 240 [g·cm^-4^·sec^-1^], TAL compliances were set to C_TALin_ = 1.5 and 0.4 [ml/mmHg] and C_TALad_ = C_TALap_ = 2.0 and 0.1 [ml/mmHg]. This high value of pulmonary resistance induces an overload in the right ventricle. By imposing such a high value, only TAL device induces some effects on the cardiopulmonary circulation. In the second step the same simulations were performed setting pulmonary arterial peripheral resistance to 120 [g·cm^-4^·sec^-1^]. This low value of pulmonary resistance (induced by drug administration) permits to download the right ventricle. By imposing such a low value, students can observe the double effects induced by TAL assistance and by the administration of drugs that improves pulmonary circulation and download the right ventricle. Finally in the third step, the same simulations were reproduced for the hybrid TAL assistance. Different combination of TAL compliances permits to evaluate the performances of the assistance [14].

In all simulations, conduced in interactive mode, the analyzed variables were: left ventricular end diastolic volume (LVEDV), left ventricular end systolic volume (LVESV), right ventricular end diastolic volume (RVEDV), right ventricular end systolic volume (RVESV), cardiac output (CO), mean pulmonary arterial pressure, mean left atrial pressure, mean systemic venous pressure and the value of the area of coronary blood flow-aortic pressure loop (CBP-AoP) [11, 33, 34]. The mean values were calculated during the cardiac cycle.

At the end of the training course, a questionnaire was submitted to students in order to evaluate the quality of the training course. The evaluation of the answers provided by students is reported in the next section. Such a precious feedback gave us an indication of the quality of CARDIOSIM^©^ and it will allow us to focus even better on users’ needs in the future releases of the tool.

## Results and discussion

**Figure 6:**
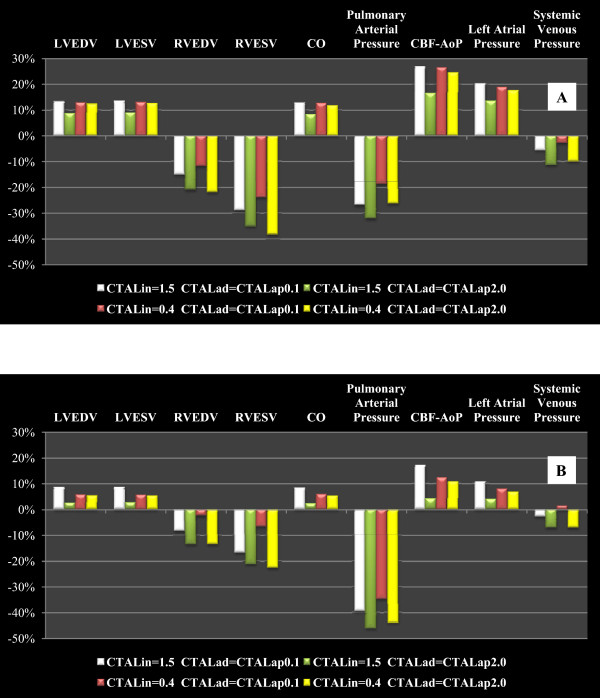
**Percentage changes produced by parallel TAL assistance (panel A) on left/** **right ventricular end diastolic volume** **(LVED/** **RVED),** **left/** **right ventricular end systolic volume** **(LVESV/** **RVESV),** **cardiac output** **(CO),** **mean pulmonary arterial pressure,** **coronary blood flow-** **aortic pressure** **(CBF-** **AoP)** **area,** **mean left atrial pressure and mean systemic venous pressure.** In panel **B** are reported the percentage changes induced by hybrid TAL assistance. These results are obtained starting from baseline conditions reproduced by measured values on hospitalized patient. The selection of the patient undergoing TAL assistance has been carried out, previously, by the students on the basis of ontological concepts based on the evaluation of haemodynamic measured parameter values. During the simulations pulmonary arterial peripheral resistance was set to 240 [g·cm^-4·^sec^-1^].

Figure 6 shows results obtained in the conditions described in the first step of “Experimental method” section. Starting from the application of baseline condition, students applied the TAL assistance in parallel and hybrid model using the numerical simulator. In Figure 6, panel A shows the percentage changes obtained applying the parallel TAL assistance and setting pulmonary arterial peripheral resistance to 240 [g·cm^-4^·sec^-1^]. TAL assistance reduces RVEDV and RVESV, but increases LVEDV and LVESV [35]. The reduction of RVEDV and RVESV allows the right ventricular pressure-volume (P-V) loop to move to the left in the pressure-volume plane [36]. According with literature data, parallel assistance can produce an increase in cardiac output [37, 38]. A reduction of mean pulmonary arterial pressure and an increase of CBF-AoP area have been produced by parallel TAL mode. In this tutorial, students have verified that parallel attachment can significantly reduce pulmonary pressures and unload the right ventricle [38, 39]. Panel B shows the effects induced by hybrid TAL assistance when peripheral resistance was set to 240 [g·cm^-4^·sec^-1^].

In both panels we reported results obtained for different values of TAL compliances. Students using different combinations of TAL compliance values verified the different effects induced on haemodynamic variables [13, 40]. The percentage changes induced by hybrid TAL assistance on some haemodynamic variables are lower than those produced by parallel assistance. Only in the case of the mean pulmonary arterial pressure, there is a percentage reduction which, in case of hybrid assistance, is greater (about 40%) than in case of parallel assistance (20-30%). Finally, students have observed that TAL assistance increases the mean left atrial pressure, as described in literature [37].

**Figure 7:**
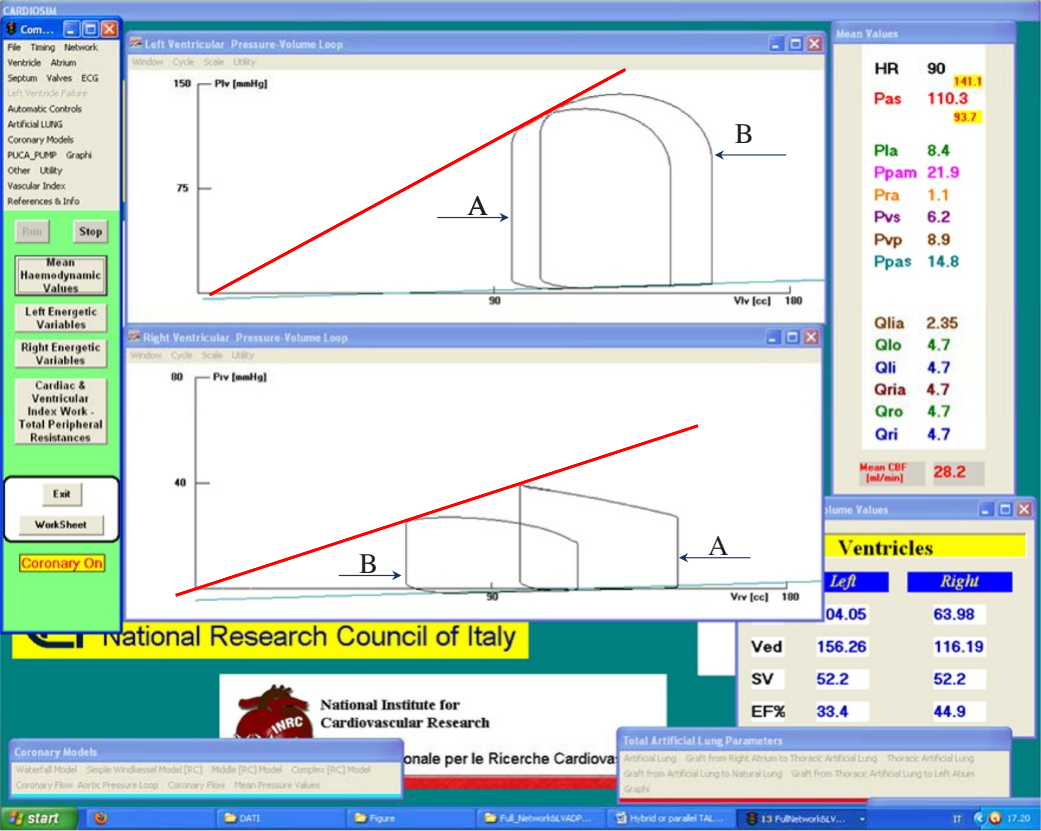
**Possible screen output produced by student during the training course using the interactive CARDIOSIM** ^**©**^ **software.** Starting from baseline conditions the applications of parallel TAL assistance induces a shift of both left (upper window) and right (lower window) P-V ventricular loops. Right P-V ventricular loop shift from **A** (baseline conditions) to **B** (assisted conditions). Left P-V ventricular loop shift (in the right side) from **A** to **B**. In the right box are reported some haemodynamic mean (calculated during the cardiac cycle) values assumed during the parallel TAL assistance.

Figure 7 shows one of the different screen outputs produced by students during the training course. In the central windows there are plotted the left ventricular (upper) and the right ventricular (lower) P-V loops [21]. In Figure 7, students reproduced the evolution of the left and right ventricular P-V loop, when TAL assistance was applied in parallel mode starting from a baseline condition. By analysing the figure, it is possible to observe how the left ventricular pressure-volume loop moves to the right (increasing LVEDV and LVESV) in the P-V plane (upper window) from position A (baseline conditions) to position B (assisted conditions). The right ventricular P-V loop (in lower window) moves towards the right side in the P-V plane from position A (baseline conditions) to position B (assisted conditions). This effect produces a reduction of RVEDV and RVESV and an increase of right ventricular efficiency.

**Figure 8:**
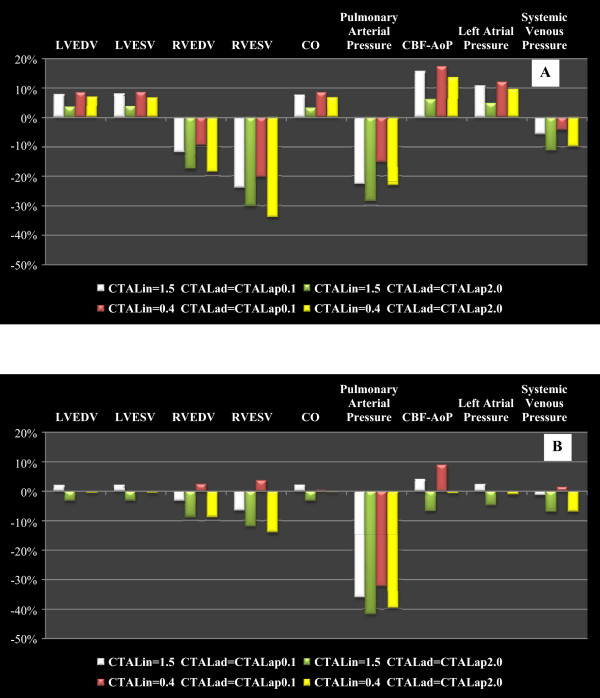
**Percentage changes produced on haemodynamic variables by TAL assistance when pulmonary arterial peripheral resistance was set to 120** **[** **g** **·** **cm** ^**-4**^ **·sec** ^**-1**^ **].** Panel **A (B)** shows the percentage changes when parallel (hybrid) TAL assistance was applied. These results are obtained starting from baseline conditions reproduced by measured values on hospitalized patient. The selection of the patient undergoing TAL assistance has been carried out, previously, by the students on the basis of ontological concepts based on the evaluation of haemodynamic measured parameter values.

Figure 8 shows the percentage changes of some haemodynamic variables when the pulmonary arterial peripheral resistance was set to 120 [g·cm^-4^·sec^-1^] in order to reproduce the effects of drug administration. Panel A shows the percentage changes when parallel TAL assistance was applied. The trend presented in Figure 8 (panel A) is the same presented in Figure 6 (panel A), but the percentage changes on CO, CBF-AoP and mean left atrial pressure are more relevant in this second figure. Percentage changes on LVESV and LVED (panel A) are higher in Figure 6 than in Figure 8. As far as hybrid TAL assistance is concerned, panel B of Figure 8 shows an opposite trend (with respect to panel B, Figure 6) for the percentage changes in some variables in correspondence with some combinations of TAL compliances value. This phenomenon occurs in LVESV, LVEDV, RVESV, RVEDV, cardiac output, mean left atrial pressure and in CBF-AoP area. The reduced pulmonary peripheral resistance value during hybrid TAL assistance apparently does not affect percentage changes in LVESV, LVEDV, CO and mean left atrial pressure.

**Figure 9:**
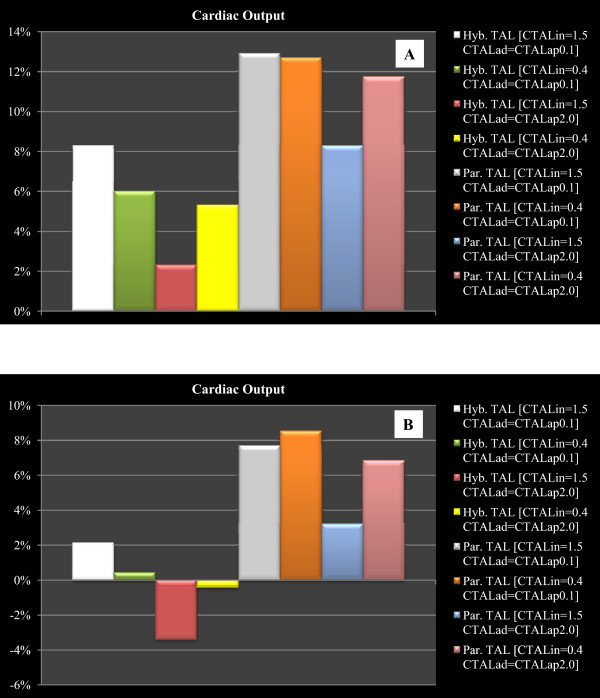
**Percentage changes produced on cardiac output by different TAL assistance when pulmonary arterial peripheral resistance was set to 240** **[** **g** **·** **cm** ^-**4**^ **·** **sec** ^**-1**^ **]** **(panel A) and to 120** **[** **g** **·** **cm** ^**-4**^ **·** **sec** ^**-1**^ **]** **(panel B).** In both panels students obtained the percentage changes setting (in the interactive human numerical simulator) different TAL compliance values. During the assistance compliances were set to C_TALin_ = 1.5 and 0.4 [ml/mmHg] and C_TALad_ = C_TALap_ = 2.0 and 0.1 [ml/mmHg].

The cardiac output is one of the variables on which researchers focus attention during TAL assistance. Results presented in Figure 9 have been also obtained during the training course held for students of the School of Specialization in Cardiology at the CRNAGS Department. In this figure we present the percentage changes (with respect to baseline conditions) of CO when TAL assistance was applied in different mode, with different pulmonary arterial peripheral resistance values and with different TAL compliance values.

Panel A (pulmonary arterial peripheral resistance was set to 240 [g·cm^-4^·sec^-1^]) emphasizes that, in the case of TAL parallel assistance, independently from the values assumed by TAL compliances, cardiac output is more or less double with respect to hybrid assistance. Panel B (pulmonary arterial peripheral resistance was set to 120 [g·cm^-4^·sec^-1^]) shows that parallel TAL assistance causes an increase in CO, whereas hybrid assistance can yield into a reduction in CO [37].

Finally, the analysis of the questionnaire submitted to students showed that:

- 80% of the students claimed to have had a good degree of learning in relation to what is explained and exercises made during the training course;
- 8% claimed to have had a sufficient degree of learning;
- 12% claimed to have had a low degree of learning.

The majority of students (88%) assessed software easy to use, while 12% of them experienced difficulties in navigating among the various alternatives offered by the simulator.

Interactive numerical simulators permit to plan for the learner exercises where he can make mistakes without jeopardizing the health of the patient or animal [41]. The same exercises can be reproduced an unlimited number of times. Additionally CARDIOSIM^©^ software allows to store specific situations in order to continue in later times the study that you are performing. The possibility to create a large number of data files from which learner can start to study the various therapies that can be administered to the virtual patient, allows to realize a database that can be indexed using an ontology of cardiovascular concepts [42–44].

During the described course, in same cardiocirculatory conditions, students applying TAL assistance induced an increase of LVEDV, LVESV and a reduction of CO. These results induced a decrease in the left ventricular efficiency. In order to improve the left ventricular conditions a possible chooses consisted to help the left ventricle with a mechanical circulatory assist device. To choose the better mechanical circulatory assist device according to the virtual patient pathological conditions, students were assisted by an ontology applied to mechanical circulatory support systems [32].

Following the same trend presented in literature data, this e-Learning experience produced the following benefits:

✓ opportunities for accelerated learning (as proved by analysing students’ questionnaires) [3];

✓ lower costs with respect to in vivo experiments;

✓ increased attention span, due to interactive nature of the tool which is an added value compared to traditional textbook learning [45];

✓ no medical related accidents [41, 46];

In general, the interactive simulator can also be used to teach and practice the management of complex perioperative medical events and emergencies [47].

The cardiovascular system is very complex and is characterized by the presence of numerous control mechanisms that can occur under certain conditions.

A limitation in the use of these simulators is linked to the impossibility to reproduce the behaviour of the entire CVS and its control mechanisms in the various circulatory conditions that may arise. Over the years, researchers have developed several numerical models of the CVS. Each model, developed in a more or less complex mode, has the characteristic to be rigid and not flexible since it was developed according to the type of study and/or experiment that the user wanted to perform [47–52].

CARDIOSIM^©^ has a different approach: it is a peculiar software platform with unique characteristics that we sketch out in the following.

First of all, unlike other *in silico* simulator [53–57], it is based on a modular approach and it is equipped with a library of numerical models of the various sections of the CVS. The library can be increased with other numerical models and can be continually updated. The various models can be easily assembled, from researchers, teachers or students, among them according to the experiment that the user wants to reproduce. This is a first feature that distinguishes CARDIOSIM^©^ from other models that despite being already available in the network, do not have the flexibility that is required to adapt to the type of pathology experiment that the user want to study.

For each set of parameters, CARDIOSIM^©^ permits to choose an initial set of values inside libraries that contains also patient or animal measured value parameters. The software simulator allows to save in Excel format the numerical values of the various waveforms reproduced during the simulations. In this way it is possible to do any kind of “off-line” analysis.

Another peculiarity of the software platform is the possibility of using several ventricular assist devices reproducing continuous or pulsatile flow. These VADs can be connected in series or in parallel mode to the left or right (or both) ventricles. VADs implemented in CARDIOSIM^©^ are pneumatic or rotary blood pump (i.e. Hemopump HP31 [9], PUCA pump [58]). Each VAD can be synchronized or not synchronized with the heart’s electrical activity or can operate in “full-empty” mode. Also intraaortic balloon pump (IABP) and biventricular pacemaker devices can be used with the numerical simulator when pathological conditions are simulated. Unlike the previously presented tools, our software platform allows to choose among different types of mechanical ventilatory assistance when VADs, IABP or biventricular pacemaker are applied or not. This option allows to study the effects produced on haemodynamic and energetic variables by the interaction among the CVS, VAD and MVA or TAL.

Some limitations concerning this training course experience (the first of such a kind for us) are described below:

- the questionnaire consisted of a small number of simple qualitative questions. In the future we intend to submit to students a more detailed questionnaire from which one can infer more analytical considerations;
- in the students’ use of the simulator, we observed they have no sufficient confidence with such kind of tools: in particular, it is very difficult for them to understand how a set of RLC elements can simulate a blood vessel;
- a limitation of our simulator lies the fact that its use is not intuitive and presents difficulties for students not familiar with IT tools.

## Conclusions

An e-Learning environment can take profit from the employment of a software simulator such as CARDIOSIM^©^.

Among the advantages of using a simulator, we have to consider that the same pathophysiological conditions can be replicated several times by the students without any damage for the virtual patient.

In particular, it is possible to simulate:

- Drug administration and their effects on haemodynamic variables
- Circulatory mechanical and ventilatory support devices
- The effects of choosing, configuring and activating one of such devices
- The effects of possible adjustments of device parameters

Currently, the e-Learning tool that we implemented is used in a local environment in a traditional face-to-face interaction between teachers and students. In the future, we plan to extend the e-Learning features in a telematic virtual environment by means of an internet based platform.

We also believe that not only simulation software, but also an ontology-based modelling of the relevant concepts involved in the e-Learning domain is a valid approach in order to reuse an environment in other domains or for other students’ profiles (e.g. general practitioners, anesthesiologists, nurses).

## References

[1] Trondsen E (2001). Games and Simulations in E-Learning. SRI Business Intelligence Consultig.

[2] Chi D, Kokkevis E, Ogunyemi O, Bindiganavale R, Hollick M, Clarke J, Webber B, Badler N, Morgan KS, Hoffman HM, Stredney D, Weghorst SJ (1997). Simulated casualties and medics for emergency training. Medicine Meets Virtual Reality.

[3] Okuda Y, Bryson EO, DeMaria S, Jacobson L, Quinones J, Shen B, Levine AI (2009). The utility of simulation in medical education: what is the evidence?. Mt Sinai J Med.

[4] Suebnukarn S, Haddawy P (2007). COMET: a collaborative tutoring system for medical problem-based learning. IEEE Intell Syst.

[5] De Lazzari C (2011). CARDIOSIM©- cardiovascular software simulator.

[6] De Lazzari C, Ferrari G, Mimmo R, Tosti G, Ambrosi D (1994). A desk top computer model of the circulatory system for heart assistance simulation: effect of an LVAD on energetic relationships inside the left ventricle. Med Eng Phys.

[7] De Lazzari C, L’Abbate A, Micalizzi M, Trivella MG, Neglia D (2014). Effects of amlodipine and adenosine on coronary haemodynamics: in vivo study and numerical simulation. Comput Meth Biomech Biomed Eng.

[8] De Lazzari C, Darowski M, Ferrari G, Pisanelli DM, Tosti G (2006). The impact of rotary blood pump in conjunction with mechanical ventilation on ventricular energetic parameters: numerical simulation. Methods Inf Med.

[9] De Lazzari C, Darowski M, Ferrari G, Pisanelli DM, Tosti G (2006). Modelling in the study of interaction of Hemopump device and artificial ventilation. Comput Biol Med.

[10] De Lazzari C, D’Ambrosi A, Tufano F, Fresiello L, Garante M, Sergiacomi R, Stagnitti F, Caldarera CM, Alessandri N (2010). Cardiac resynchronization therapy: could a numerical simulator be a useful tool in order to predict the response of the biventricular pacemaker synchronization?. Eur Rev Med Pharmacol Sci.

[11] De Lazzari C, Del Prete E, Genuini I, Fedele F (2013). In silico study of the haemodynamic effects induced by mechanical ventilation and biventricular pacemaker. Comput Methods Prog Biomed.

[12] Perme SC, Southard RE, Joyce DL, Noon GP, Loebe M (2006). Early mobilization of LVAD recipients who require prolonged mechanical ventilation. Tex Heart Inst J.

[13] McGillicuddy JW, Chambers SD, Galligan DT, Hirschl RB, Bartlett RH, Cook KE (2005). In vitro fluid mechanical effects of thoracic artificial lung compliance. ASAIO J.

[14] Boschetti F, Perlman CE, Cook KE, Mockros LF (2000). Hemodynamic effects of attachment modes and device design a thoracic artificial lung. ASAIO J.

[15] Colquitt RB, Colquhoun DA, Thiele RH (2011). In silico modeling of physiologic systems. Best Pract Res Clin Anaesthesiol.

[16] Sinz E (2005). Simulation-based education for cardiac, thoracic, and vascular anesthesiology. Semin Cardiothorac Vasc Anesth.

[17] Wildhaber RA, Verrey F, Wenger RH (2011). A graphical simulation software for instruction in cardiovascular mechanics physiology. Biomed Eng Online.

[18] Zwischenberger JB, Anderson CM, Cook KE, Lick SD, Mockros LF, Bartlett RH (2001). Development of an implantable artificial lung: challenges and progress. ASAIO J.

[19] Federspiel WJ, Svitek RG, Wnek GE, Bowlin GL (2004). Artificial lungs: current research and future directions. Encyclopedia of Biomaterials and Biomedical Engineering.

[20] Maughan WL, Sunagawa K, Sagawa K (1987). Ventricular systolic interdependence: volume elastance model in isolated canine hearts. Am J Physiol Heart Circ Physiol.

[21] Sagawa K, Maughan WL, Suga H, Sunagawa K (1988). Cardiac Contraction and the Pressure-Volume Relationships.

[22] Korakianitis T, Shi Y (2006). A concentrated parameter model for the human cardiovascular system including heart valve dynamics and atrioventricular interaction. Med Eng Phys.

[23] De Lazzari C (2012). Interaction between the septum and the left (right) ventricular free wall in order to evaluate the effects on coronary blood flow: numerical simulation. Comput Meth Biomech Biomed Eng.

[24] Starling EH (1918). The Linacre Lecture on the Law of the Heart.

[25] Guyton AC, Jones CE, Coleman TG (1973). Computer analysis of total circulatory function and of cardiac output regulation. Circulatory Physiology: Cardiac Utput and its Regulation.

[26] Frasch HF, Kresh JY, Noordergraaf A (1996). Two-port analysis of microcirculation: an extension of windkessel. Am J Physiol.

[27] Shi Y, Lawford P, Hose R (2011). Review of zero-D and 1-D models of blood flow in the cardiovascular system. BioMed Eng Online.

[28] Downey JM, Kirk ES (1975). Inhibition of coronary blood flow by a vascular waterfall mechanism. Circ Res.

[29] Spaan JA, Nreuls NP, Laird JD (1981). Diastolic–systolic coronary flow differences are caused by intramyocardial pump action in the anesthetized dog. Circ Res.

[30] Spaan JA, Nreuls NP, Laird JD (1981). Forward coronary flow normally seen in systole is the result of both forward and concealed back flow. Basic Res Cardiol.

[31] De Lazzari C, Darowski M, Ferrari G, Clemente F, Guaragno M (2001). Ventricular energetics during mechanical ventilation and intraaortic balloon pumping – Computer simulation. J Med Eng Technol.

[32] De Lazzari C, Guerrieri E, Pisanelli DM, Murray A (2003). A domain ontology for mechanical circulatory support systems. Proceeding of the 30TH Annual Conference of Computers in Cardiology: 21–24 Sept. 2003.

[33] Di Mario C, Kramas R, Gil R, Serruys PW (1994). Slope of the instantaneous hyperemic diastolic CFV-pressure relation. A new index for assessment of the physiological significance of coronary stenosis in humans. Circulation.

[34] Krams R, Ten Cate FJ, Carlier SG, van der Steen AFW, Serruys PW (2003). Diastolic coronary vascular reserve: a new index to detect changes in the coronary microcirculation in hypertrophic cardiomyopathy. J Am Coll Cardiol.

[35] Lick SD, Zwischenberger JB, Wang D, Deyo DL, Alpard SK, Chambers SD (2001). Improved right heart function with a compliant inflow artificial lung in series with the pulmonary circulation. Ann Thorac Surg.

[36] Haft JW, Montoya P, Alnajjar O, Posner SR, Bull LL, Iannettoni MD, Bartlett RH, Hirschl RB (2001). An artificial lung reduces pulmonary impedance and improves right ventricular efficiency in pulmonary hypertension. J Thorac Cardiovasc Surg.

[37] Perlman CE, Cook KE, Seipelt R, Mavroudis C, Backer CL, Mockros LF (2005). In vivo hemodynamic responses to artificial lung attachment. ASAIO J.

[38] Akay B, Reoma JL, Camboni D, Pohlmann JR, Albert JM, Kawatra A, Gouch AD, Bartlett RH, Cook KE (2010). In-parallel artificial lung attachment at high flows in normal and pulmonary hypertension models. Ann Thorac Surg.

[39] Akay B, Foucher JA, Camboni D, Koch KL, Kawatra A, Cook KE (2012). Hemodynamic design requirements for in series thoracic artificial lung attachment in a model of pulmonary hypertension. ASAIO J.

[40] Haft J, Bull JL, Rose R, Katsra J, Grotberg JB, Bartlett RH, Hirschl RB (2003). Design of an artificial lung compliance chamber for pulmonary replacement. ASAIO J.

[41] Ahlberg G, Enochsson L, Gallagher AG, Hedman L, Hogman C, McClusky DA 3rd, Ramel S, Smith CD, Arvidsson D (2007). Proficiency-based virtual reality training significantly reduces the error rate for residents during their first 10 laparoscopic cholecystectomies. Am J Surg.

[42] Backhaus M, Chung JD, Cowan BR, Tao W, Young AA, Kerckhoffs R (2010). The cardiac atlas project: towards a map of the heart. Patient-Specific Modeling of the Cardiovascular System, Volume 1.

[43] Eccher C, Scipioni A, Miller AA, Ferro A, Pisanelli DM (2013). An ontology of cancer therapies supporting interoperability and dataconsistency in EPRs. Comput Biol Med.

[44] Eccher C, Purin B, Pisanelli DM, Battaglia M, Apolloni I, Forti S (2006). Ontologies supporting continuity of care: the case of heart failure. Comput Biol Med.

[45] Wayne DB, Didwania A, Feinglass J, Fudala MJ, Barsuk JH, McGaghie WC (2008). Simulation-based education improves quality of care during cardiac arrest team responses at an academic teaching hospital: a case–control study. Chest.

[46] Vincent C, Moorthy K, Sarker SK, Chang A, Darzi AW (2004). Systems approaches to surgical quality and safety: from concept to measurement. Ann Surg.

[47] Chakravarthy B, ter Haar E, Bhat SS, McCoy CE, Denmark TK, Lotfipour S (2011). Simulation in medical school education: review for emergency medicine. Western J Emerg Med.

[48] Heldt T, Shim EB, Kamm RD, Mark RG (2002). Computational modeling of cardiovascular response to orthostatic stress. J Appl Physiol.

[49] Mukkamala R, Cohen RJ (2001). A forward model-based validation of cardiovascular system identification. Am J Physiol Heart Circ Physiol.

[50] Mukkamala R, Kim JK, Li Y, Sala-Mercado J, Hammond RL, Scislo TJ, O’Leary DS (2006). Estimation of arterial and cardiopulmonary total peripheral resistance baroreflex gain values: validation by chronic arterial baroreceptor denervation. Am J Physiol Heart Circ Physiol.

[51] Sheffer L, Santamore WP, Barnea O (2007). Cardiovascular simulation toolbox. Cardiovasc Eng.

[52] Brunberg A, Heinke S, Spillner J, Autschbach R, Abel D, Leonhardt S (2009). Modeling and simulation of the cardiovascular system: a review of applications, methods, and potentials. Biomed Tech (Berlin).

[53] Goldberger AL, Amaral LAN, Glass L, Hausdorff JM, Ivanov PC, Mark RG, Mietus JE, Moody GB, Peng CK, Stanley HE (2000). PhysioBank, PhysioToolkit, and PhysioNet: components of a new research resource for complex physiologic signals. Circulation.

[54] Bassingthwaighte JB (2000). Strategies for the physiome project strategies for the physiome project. Ann Biomed Eng.

[55] Garny A, Nickerson DP, Cooper J, dos Santos RW, Miller AK, McKeever S, Nielsen PMF, Hunter PJ (2008). CellML and associated tools and techniques. Philos Trans A Math Phys Eng Sci.

[56] Lian J (2010). Open source modeling of cardiovascular system: a brief overview. TOPETJ.

[57] Barnea O (2010). Open-source programming of cardiovascular pressure-flow dynamics using SimPower toolbox in Matlab and Simulink. Open Pacing Electrophysiol Ther J.

[58] De Lazzari C, Genuini I, Quatember B, Fedele F (2014). Mechanical ventilation and thoracic artificial lung assistance during mechanical circulatory support with PUCA pump: in silico study. Comput Methods Programs Biomed.

